# Cells with hematopoietic potential reside within mouse proepicardium

**DOI:** 10.1007/s00418-018-1661-1

**Published:** 2018-03-16

**Authors:** Ewa Jankowska-Steifer, Justyna Niderla-Bielińska, Bogdan Ciszek, Marek Kujawa, Mateusz Bartkowiak, Aleksandra Flaht-Zabost, Daria Klosinska, Anna Ratajska

**Affiliations:** 1 Department of Histology and Embryology, Center for Biostructure, Medical University of Warsaw, Chalubińskiego 5, 02-004 Warsaw, Poland; 2 Department of Anatomy, Medical University of Warsaw, Warsaw, Poland; 3 Department of Pathology, Medical University of Warsaw, Warsaw, Poland; 4 Department of Histology and Embryology, Warsaw University of Life Sciences, WULS, SGGW Nowoursynowska 166, 02-787 Warsaw, Poland

**Keywords:** Hematopoiesis, Proepicardium, Hematopoietic precursor cells, Hemogenic endothelium

## Abstract

During embryonic development, hematopoietic cells are present in areas of blood-vessel differentiation. These hematopoietic cells emerge from a specific subpopulation of endothelial cells called the hemogenic endothelium. We have previously found that mouse proepicardium contained its own population of endothelial cells forming a network of vascular tubules. We hypothesize that this EC population contains cells of hematopoietic potential. Therefore, we investigated an in vitro hematopoietic potential of proepicardial cell populations. The CD31^+^/CD45^−^/CD71^−^ cell population cultured for 10 days in MethocultTM gave numerous colonies of CFU-GEMM, CFU-GM, and CFU-E type. These colonies consisted of various cell types. Flk-1^+^/CD31^−^/CD45^−^/CD71^−^, and CD45^+^ and/or CD71^+^ cell populations produced CFU-GEMM and CFU-GM, or CFU-GM and CFU-E colonies, respectively. Immunohistochemical evaluations of smears prepared from colonies revealed the presence of cells of different hematopoietic lineages. These cells were characterized by labeling with various combinations of antibodies directed against CD31, CD41, CD71, c-kit, Mpl, Fli1, Gata-2, and Zeb1 markers. Furthermore, we found that proepicardium-specific marker WT1 co-localized with Runx1 and Zeb1 and that single endothelial cells bearing CD31 molecule expressed Runx1 in the proepicardial area of embryonic tissue sections. We have shown that cells of endothelial and/or hematopoietic phenotypes isolated from mouse proepicardium possess hematopoietic potential in vitro and in situ. These results are supported by RT-PCR analyses of proepicardial extract, which revealed the expression of mRNA for crucial regulatory factors for hemogenic endothelium specification, i.e., Runx1, Notch1, Gata2, and Sox17. Our data are in line with previous observation on hemangioblast derivation from the quail PE.

**Electronic supplementary material:**

The online version of this article (10.1007/s00418-018-1661-1) contains supplementary material, which is available to authorized users.

## Introduction

During fetal life, hematopoiesis begins at 7.5 dpc (day post coitum) in blood islands located in the endodermal wall of the extraembryonic yolk sac (Palis et al. 1999; Palis and Yoder 2001). First, intraembryonic blood cell formation is detectable at around 10.5 dpc in the aorta/gonad/mesonephros (AGM) region (Matsuoka et al. 2001).

Numerous observations indicate that clusters of hematopoietic cells are present in various areas in which embryonic blood vessels differentiate. These hematopoietic cells arise from a specific subpopulation of endothelial cells (EC) called the hemogenic endothelium (HE) (Dzierzak and Speck 2008; Bertrand et al. 2010; Hirschi 2012), which is capable of endothelial-to-hematopoietic transition (EHT) (Kissa and Herbomel 2010; Vargel et al. 2016). The presence of HE and the EHT occurrence during early period of development have been demonstrated in the ventral wall of the dorsal aorta at the level of the developing gonad/mesonephros, in the placenta, in the vitelline and umbilical arteries, as well as in the embryonic head and the somites (de Bruijn et al. 2000; Gekas et al. 2005; Li et al. 2012; Antas et al. 2013; Nakano et al. 2013; Yzaguirre and Speck 2016). Hemogenic activity can also be detected in some endothelial cells in the endocardium area located in the outflow tract cushions and the atria of 8.25–8.5 dpc-old hearts (Nakano et al. 2013).

It has been hypothesized that fetal hematopoiesis originates from hemangioblasts and/or from the hemogenic endothelium. Hemangioblasts, the common precursors of hematopoietic and endothelial lineages, are generated in the primitive streak; these cells transiently appear during prenatal development (Choi 1998; Ciau-Uitz and Patient 2016) and express the T-box transcription factor Brachyury (T) and the fetal liver kinase receptor (Flk1). A second hypothesis assumes that hematopoietic cell lineage arises in result of de-differentiation of endothelial cells (Jaffredo et al. 1998). Hemogenic endothelial cells express common markers for endothelial and hematopoietic cells, such as CD31, VE cadherin, Flk1, c-kit, CD41, and Sca-1 (Zovein et al. 2008; Boisset et al. 2010). Moreover, the transcription factor Runx1, a key hematopoietic regulator, is expressed by endothelial cells in hemogenic vascular sites (Tober et al. 2016; Yzaguirre and Speck 2016; de Bruijn and Dzierzak 2017). Recently, it has been demonstrated that Runx1 is also expressed in non-hemogenic endothelial cells of mouse embryos and that it promotes erythromyeloid progenitor formation in ectopic sites, specifically in the yolk sac, the dorsal aorta, and the heart. However, this property of Runx1 is only active before embryonic day 8.5 (Yzaguirre et al. 2018). As there is no single cellular marker for both subtypes of EC, which could distinguish the hemogenic endothelium from the non-hemogenic endothelium, several markers could be used simultaneously for this purpose. It has been suggested that the two hypotheses describing the embryonic origin of hematopoietic cells, which were regarded as mutually exclusive for many years could actually be combined, and hemangioblasts may be linked to the hematopoietic cell progeny via the HE (Lancrin et al. 2009; Antas et al. 2013).

However, it is still not clear if the entire hematopoietic activity during the embryonic period of development arises from the HE, or there are some progenitor cells for blood cells formation which are different from the HE cells. Recently, it has been proposed that blood cells precursors can also be produced by hemogenic angioblasts. Therefore, it is possible that scattered, blood-vessel-independent hemogenic progenitors may also exist (Tanaka et al. 2014; Jankowska-Steifer et al. 2015; Lacaud and Kouskoff 2017; Zamir et al. 2017).

The proepicardium (PE) is a small, transient structure essential to heart development, which appears on the venous pole of murine embryonic heart from 9.0 dpc to 9.75 dpc (Schulte et al. 2007; Ratajska et al. 2008). The PE provides cells for the epicardium, and it regulates the development of the myocardial wall and coronary blood vessels. It has been demonstrated that some epicardium-derived cells differentiate into fibroblasts, smooth muscle cells, some coronary endothelial cells, and presumably some cardiac myocytes. It has also been documented that some of the erythroblasts/erythrocytes located in blood islands and small vascular tubes of quail embryos differentiated from hemangioblasts and that the latter derived from the proepicardium (Tomanek 2006). The PE is composed of mesenchymal cells expressing various transcription factors (e.g., WT1, Tbx18, and Tcf21). Recently, PE endothelial cells have been investigated immunohistochemically by means of antibodies directed against a variety of cellular markers, including CD31, Flk-1, Lyve-1, and Tie-2 (Cossette and Misra 2011; Niderla-Bielinska et al. 2015). Immature endothelial cells form proepicardial capillary network, which is continuous with the sinus venosus (Niderla-Bielińska et al. 2015).

Because the mouse proepicardium provides progenitor cells for the developing heart, and also is endowed with its own endothelium-lined capillary system, it could be considered as a potential source of hemangioblasts/hemogenic endothelium. Hence, we believe that in mouse, the PE could have a hematopoietic potential. Our hypothesis is supported by the finding that both the PE and HE originate from the lateral plate mesoderm (Zovein et al. 2010; Maya-Ramos et al. 2013).

This research project was aimed at investigating if selected populations of endothelial cells and non-endothelial mesenchymal cells, isolated from the PE, have a hematopoietic potential when cultured in vitro. This study was carried out by means of a magnetic-activated cell sorting system (MACS) (used for the isolation of endothelial and hematopoietic cell populations from the PE), a colony-forming cell (CFC) assay (used for evaluation of cell types cultured in vitro), and a laser confocal microscopy of immunohistochemically stained cells labeled with antibodies against different hematopoietic lineages markers. We also employed RT-PCR to assess the mRNA expression of some transcription factors crucial for the HE formation, e.g., Notch1, Gata2, Runx1, Sox17, Nkx2-5. Our study is the first to demonstrate that the mammalian PE contains specific-cell populations which in vitro exhibit hematopoietic potential as evidenced by the colony-forming cell assay and by in situ mRNA expression for genes specific for the HE.

## Materials and methods

### Animals and PE isolation

The experiments were performed with F1 cross of C57BL/6 and CBA mouse inbred strains. One hour after mating, the presence of the vaginal plug was assessed, and that day was designated as 0 dpc. Embryos were collected at 9.5 dpc (22–25 somites stage). Mice were euthanized, and after immediate excision, embryos-containing uteri were placed in a culture dish with sterile Tris-Tyrode solution. Under an inverted microscope, embryos were excised from the uteri, devoid of fetal membranes, and positioned on the left side for better visualization of the PE. Proepicardium isolation was performed according to a method described earlier (Garriock et al. 2014). Briefly, after the removal of the pericardial sac, the embryos were cut in half just below the heart tube, and then, the PE was excised with a single cut made with fine scissors. The obtained PEs were washed in an M199 medium supplemented with 1% FCS (Fetal Calf Serum), 1% ITS (Insulin–Transferrin–Selenium), and 1% antibiotic/antimycotic solution (all purchased from Gibco®, ThermoFisher Scientific, MA, USA). For each experiment, the number of embryos originating from different litters varied from 6 to 8.

To ensure that the study fulfilled ethical guidance for research, approval was sought and obtained through the Second (II) Local Ethics Committee at the Medical University of Warsaw, Poland.

### Single-cell suspension and separation of cell subpopulations

**Scheme 1:**
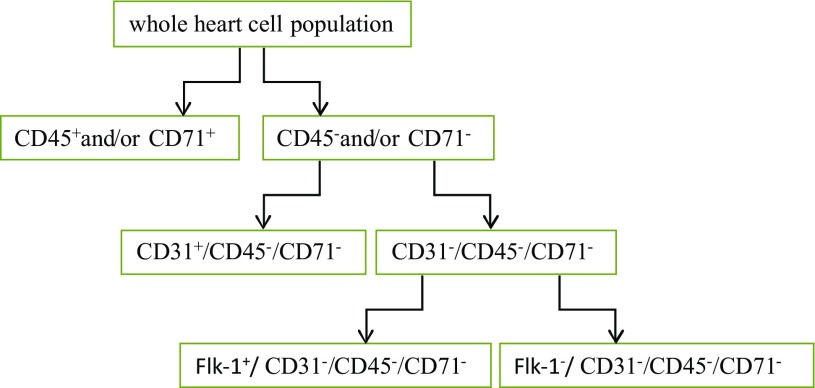
Steps involved in isolating cell subpopulations from the PE via a magnetic-activated cell sorting system (MACS)

The proepicardia were washed with Ca^2+^- and Mg^2+^-free PBS (Gibco^®^, ThermoFisher Scientific, MA, USA) and digested with accutase (Sigma-Aldrich, MI, USA) on a magnetic stirrer at room temperature for 15 min. PE tissue fragments were intensively pipetted, and if still not fully dissociated, digested for additional 10 min with accutase. Isolated cells were washed in a PBS solution containing 10% FCS (Fetal Calf Serum, Gibco^®^, ThermoFisher Scientific, MA, USA), and then transferred through a nylon filter membrane with pore size of 40 µm. Using a magnetic-activated cell sorting system (MACS Milteney Biotec Inc, CA, USA), cells were separated into different populations with indirect magnetic labeling, following the manufacturer’s instructions. In brief, the cells were first incubated with primary rat antibodies against CD31 (550,274, BD Pharmingen^™^), CD71 (553,264, BD Pharmingen^™^), CD45 (550,539, BD Pharmingen^™^), and Flk1 (555,307, BD Pharmingen^™^). Next, they were incubated with secondary anti-rat antibodies conjugated with microbead (130-048-501, Milteneyi Biotec, Inc, CA, USA). After labeling with the antibodies, the cells were separated with MACS Columns and Separators. First, the cells were subdivided into two populations: CD45/CD71 double-positive, and CD45/CD71 double-negative. Subsequently, CD31-positive and CD31-negative cells were isolated from the latter population. Finally, Flk-1-positive cells were separated from CD31-negative cells. In this way, the following cell populations were obtained and used for further procedures: Flk-1^+^/CD31^−^/CD45^−^/CD71^−^; Flk-1^−^/CD31^−^/CD45^−^/CD71^−^; CD31^+^/CD45^−^/CD71^−^; and CD45^+^ and/or CD71^+^. These steps in cell subpopulations isolation are shown in Scheme 1.

### Colony-forming cell (CFC) assay

The separated cell populations were suspended in Iscove’s MDM with 2% FBS and cultured in Methocult^™^ GF M3434 (STEMCELL Technologies, B.C., Canada) on 24-well culture plates. No additional exogenous cytokines were added to the culture medium. Cultures were maintained in an incubator in a controlled atmosphere with a temperature of 37 °C, 5% CO2 concentration, and 95% relative humidity. The colonies were identified using an inverted microscope, and inspected at day 3, 5, and 7. At day 10, different types of colonies growing in the culture were counted, and the cells from these colonies were collected for smear preparation. Those cells were either stained with Giemsa, or immunostained with antibodies directed against a variety of antigens specific for hematopoiesis.

### Immunohistochemical staining and confocal microscopy of smears

The collected cell smears were dried for 30 min at 36 °C, and then fixed with 4% buffered paraformaldehyde (PPH STANDARD, Poland) in PBS (Gibco^®^, ThermoFisher Scientific, MA, USA) at room temperature for 20 min. Next, the smears were washed with PBS and incubated with 1% BSA, 0.1% TritonX-100, and 0.1 glycine in PBS for 30 min. Then, for 1 h, the smears were labeled with a mixture of two or three primary antibodies, directed against different antigens, diluted in PBS containing 1% BSA. Primary antibodies used for labeling are listed in Table 1.

**Table 1:** Primary antibodies used and their sources

Antibodies against	Host	Final concentration	Company
CD41	Rat	1:50	Abcam, UK
Mpl	Rabbit	1:100	EMD Millipore, MA, USA
c-kit	Rat	1:50	Novous Biologicals, UK
CD71	Rat	1:30	BD Pharmingen^™^ CA, USA
Gata2	Rabbit	1: 100	Acris Antibodies GmbH, Germany
Zeb1	Rabbit	1:100	LifeSpain BioSciences, WA, USA
Fli1	Rabbit	1:800	LifeSpain BioSciences, WA, USA
WT1	Mouse	1:30	DAKO, Glostrup, Denmark
CD31	Rat	1:100	BD Pharmingen^™^, CA, USA
Runx1	Rabbit	1:200	Thermo Fisher Scientific, MA, USA
Notch1	Mouse	1:100	Abcam, UK

Subsequently, after three washes in PBS, the cells were incubated with secondary donkey antibodies at room temperature for 1 h. Then, the smears were washed in PBS. The following secondary antibodies conjugates were used: Cy3^TM^anti-rabbit IgG, Alexa Fluor 647 anti-rat IgG, or FITC-anti-mouse IgG (Jackson ImmunoResearch Laboratories, ME, USA). Immunohistochemical negative control sections were prepared using the same, previously described procedure, except that the primary antibodies were missing and replaced by PBS with 1% BSA.

Next, to visualize cell nuclei, the smears were stained with DAPI (Thermo Fisher Scientific, MA, USA) at room temperature for 2 min, and then mounted with DAKO immunofluorescent medium (Dako, Denmark). A Leica confocal microscope-type TCS SP5 was used for cell smears analysis. Basic system settings were: objective: Leica HC PL APO ×20/0.7; fluorescence settings for DAPI: UV 405 diode 12%, PMT1 detector, smart offset − 40%, pinhole 132, smart gain 1250, filter—substrate; Cy3^™^: HeNe 543 laser 94%, PMT2 detector, smart offset − 40%, pinhole 152, smart gain 1033, filter—TD488/543/633; FITC: Argon laser 94%, PMT3 detector, smart offset − 40%, pinhole 148, smart gain 1028, filter—TD488/543/633; C: Dylight^™^: HeNe 633 laser 37%, PMT3 detector, smart offset − 40%, pinhole 132, smart gain 962, filter—TD488/543/633.

### Giemsa staining of smears

First, the smears were fixed in 100% methanol for 30 min, next rinsed in tap water, and then treated with a freshly made solution of 10% Giemsa stain (Kolchem, Poland) for 30 min.

### Immunofluorescence staining of frozen sections of 9.5 dpc embryos

Immediately after collecting, the 9.5 dpc embryos were fixed in 4% buffered paraformaldehyde for 30 min and processed through 10–20–30% sucrose. Then, they were embedded in Tissue Freezing Medium (Leica Microsystem, Germany) and frozen in liquid nitrogen. The frozen embryos were cut serially into 10 µm sections. The sections were washed with PBS, and subsequently incubated with 1% BSA, 0.1% TritonX-100, and 0.1 glycine in PBS for 30 min. After the incubation, the sections were blocked with 5% donkey serum (Jackson ImmunoResearch Laboratories, ME, USA) for 1 h. The following primary antibodies, diluted in PBS/1% BSA, were used for labeling at room temperature for 1 h: anti-WT1, anti-CD31, anti-Runx1, and anti-Zeb1 (as presented in Table 1). After being washed three times with PBS, the slides were subsequently incubated for 1 h (as above) with secondary donkey antibodies. The secondary antibodies, conjugated with respective fluorochromes, were diluted in a PBS/1% BSA solution. Staining with DAPI and further procedures were performed as described previously for cell smears.

### Total RNA isolation, reverse transcription (RT), and Real-Time PCR

PE (9.5 dpc) and liver tissue fragments (13.5 dpc) were used for RNA isolation. RNA was isolated with NucleoSpin^®^RNA II kit (Macherey–Nagel, Duren, Germany) according to the producer’s protocol. The quality and concentration of RNA were assessed with NanoDrop spectrophotometer. The RT was performed with High Capacity RNA-to-cDNA Kit, according to the producer’s protocol (Applied Biosystems, ThermoFisher Scientific, MA, USA). The cDNA was stored at − 20 °C. Gene expression was measured with the relative quantitation (RQ) using comparative CT assay (Livak and Schmittgen 2001). Real-Time PCR was performed in Abi Prism 7500 (Applied Biosystems, ThermoFisher Scientific, MA, USA) on 96-well optical plates. Each sample was run in triplicates and supplied with endogenous control (mouse GAPDH no. Mm99999915_g1). For gene expression, the following TaqMan Expression Assays were used: for Notch1: Mm00627185_m1; for Runx1: Mm01213404_m1; for Sox17: Mm00488363_m1; for Gata2: Mm00492301_m1, and for Nkx2-5: Mm01309813_s1. All probes were stained with FAM (all purchased from Applied Biosystems, ThermoFisher Scientific, MA, USA). Reactions were run in 20 µl volume with TaqMan Universal Master Mix (Applied Biosystems, ThermoFisher Scientific, MA, USA), appropriate primer set, MGB probe, and 5 ng of cDNA template. Universal thermal conditions were used (10 min at 95 °C, 40 cycles of 15 s at 95 °C, and 1 min at 60 °C). Data analysis was done with sequence detection software version 1.2 (Applied Biosystems, ThermoFisher Scientific, MA, USA).

### Statistical analysis

The statistical analysis was performed with Statistica software ver. 12 (StatSoft, Tulsa, OK, USA). Descriptive data are presented as means and standard deviations obtained from six experiments. To compare parameters between groups, the Mann–Whitney–Wilcoxon test was used, with the level of significance set at 0.05.

## Results

**Fig. 1:**
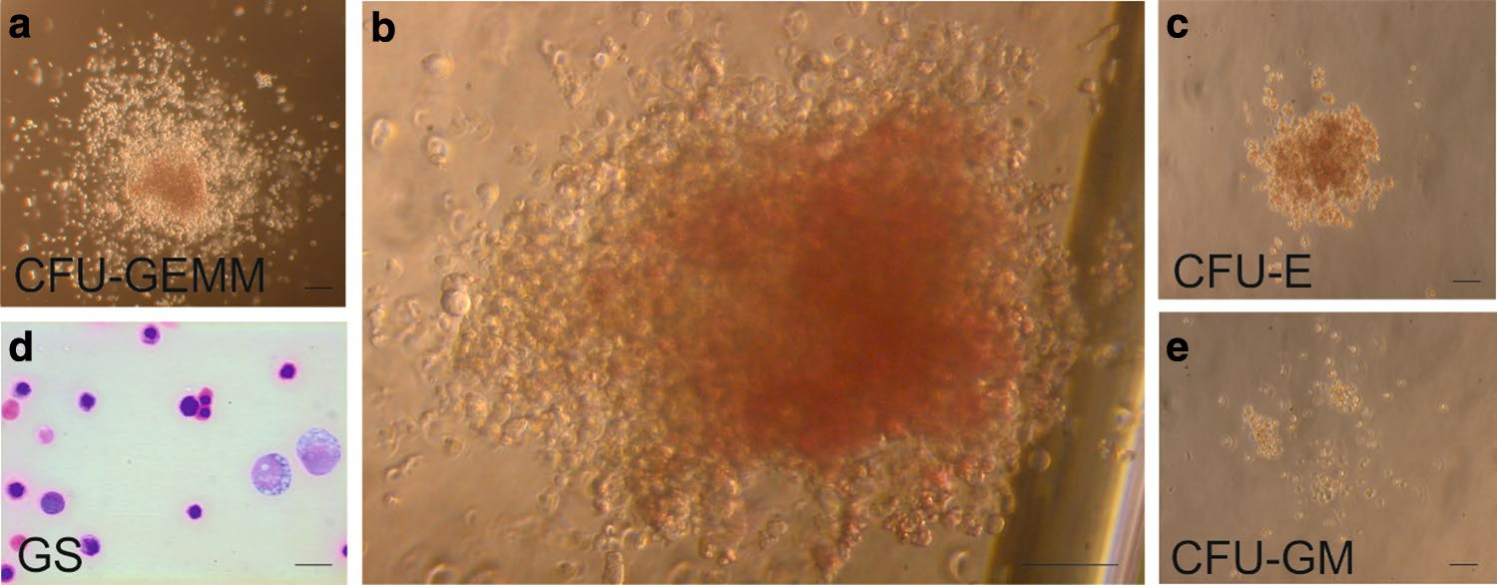
Cultured proepicardium-derived cells give rise to hematopoietic colonies. Colonies obtained from CD31+/CD45-/CD71 cells after 10 days of culture in MethoCult GF3434. Inverted microscopy of CFU-GEMM (**a, b**), CFU-E (**c**), and CFU-GM (**e**). Smear of cells aspirated from a CFU-GEMM colony stained with Giemsa (**d**). Scale bars 250 (**a**–**c, e**) and 20 µm (**d**). Lens magnification ×10/0.22 for **a, c, d, e** and ×20/0.25 for **b**

The number of colonies grown from our selected cell populations was variable (see Table 2). The highest number of colonies was obtained from the CD31^+^/CD45^−^/CD71^−^ cell population. These colonies were of different types, such as: CFU-GEMM (i.e., containing granulocytes, erythrocytes, monocytes/macrophages, and megakaryocytes; Fig. 1a, b), CFU-GM (i.e., containing granulocytes and macrophages; Fig. 1e), and CFU-E (i.e., containing erythrocytes; Fig. 1c).

**Table 2:** Number of different types of colonies grown from isolated cell populations. Data are presented as a total number of colonies obtained in five experiments

Isolated cell populations cultured in Methocult GF3434	Type of colonies		
	CFU-GEMM	CFU-GM	CFU-E
CD31^+^/CD45^−^/CD71^−^	6	4	4
Flk1^+^/CD31^−^/CD45^−^/CD71^−^	2	1	0
Flk1^−^/CD31^−^/CD45^−^/CD71^−^	0	0	0
CD45^+^ and/or CD71^+^	0	4	1

A lower number of colonies were obtained with cells of Flk1^+^/CD31^−^/CD45^−^/CD71^−^ phenotype, and those colonies were of CFU-GEMM and CFU-GM type. Two types of colonies were obtained from CD45^+^ and/or CD71^+^ cells (CFU-GM and CFU-E colonies).

Quadruple-negative cells, Flk1^−^/CD31^−^/CD45^−^/CD71^−^, did not form any colonies.

Giemsa-stained cellular smears confirmed the classification of different types of colonies, based on their morphology (Fig. 1d).

**Fig. 2:**
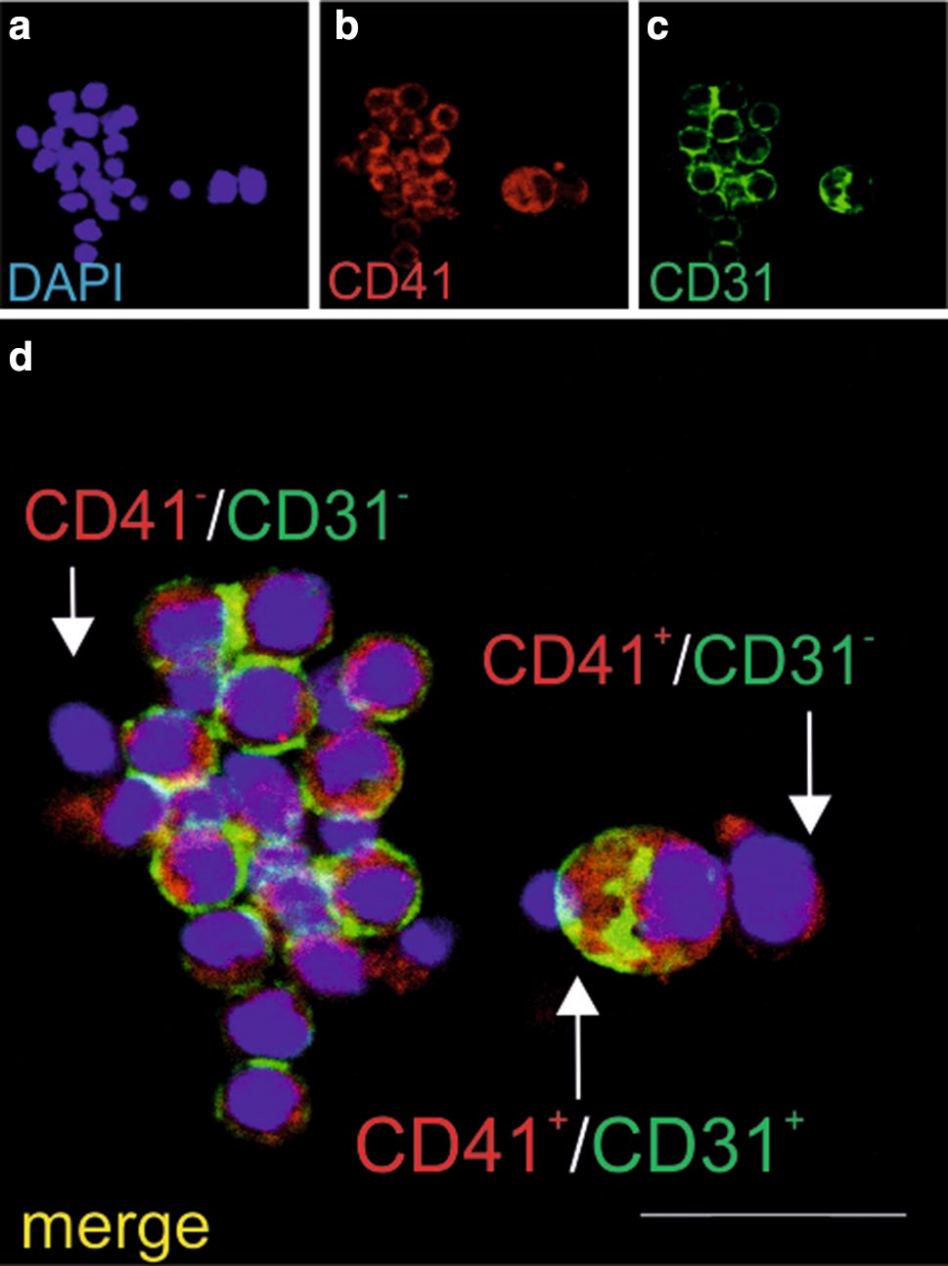
Cells growing in CFU-GEMM colonies co-express the CD31 and CD41 markers. Confocal microscope images of smears prepared from CFU-GEMM colonies grown from CD31+/CD45−/CD71− cells stained with anti-CD31 (green) (**c, d**) and anti-CD41 (red) (**b, d**) antibodies. Cell nuclei are counterstained with DAPI (blue) (**a, d**). Almost all cells are CD31/ CD41 double-positive (yellow arrow); only a few cells are CD41+/CD31− (red arrow) or CD31−/CD41− (white arrow). Scale bar 25 µm. Lens magnification ×20; zoom ×7.6

An immunohistochemical evaluation of the smears prepared from the CFU-GEMM colonies (which originated from the CD31^+^/CD45^−^/CD71^−^ cell fraction) revealed a heterogeneous cell population: numerous CD41^+^/CD31^+^ cells, and some CD41^+^ /CD31^−^ or CD41^−^ /CD31^−^ cells (Fig. 2a–d).

**Fig. 3:**
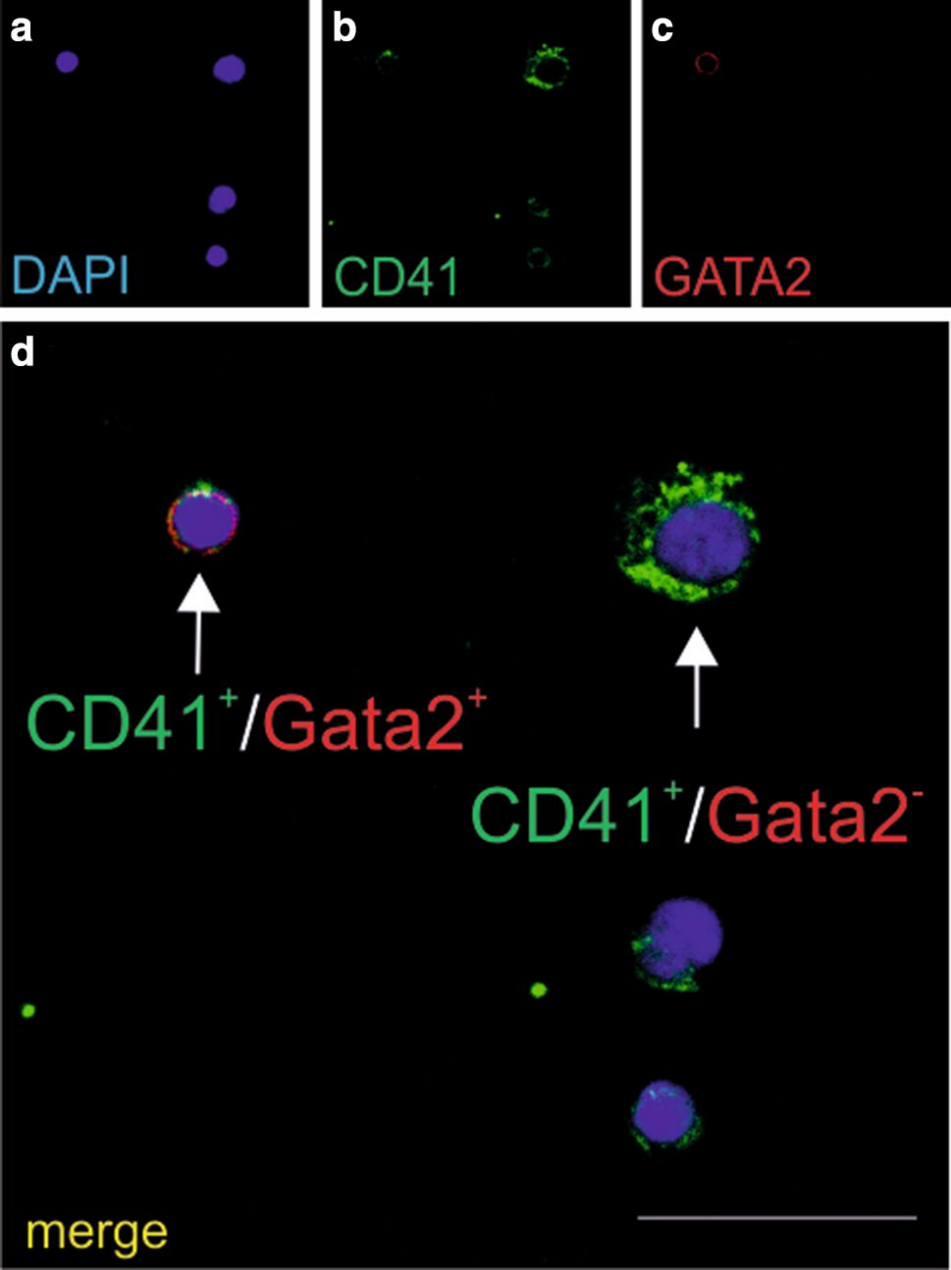
Cells growing in CFU-GEMM colonies express both CD41 and Gata2 markers. Confocal microscope images of smears prepared from CFU-GEMM colonies grown from CD31+/CD45−/CD71− cells stained with anti-CD41 (green) (**b, d**) and/or anti-Gata2 (red) (**c, d**) antibodies. Cell nuclei are counterstained with DAPI (blue) (**a, d**). All cells express the CD41 marker. Merged images (**d**) demonstrate Gata2 expression diversity. CD41+/Gata2 + cells (red arrow) and CD41+/Gata2-cells (green arrow) are present. Scale bar 25 µm. Lens magnification ×20; zoom ×7.0

Most cells expressing the CD41 marker were negative for Gata2, a transcription factor essential for early stage erythropoiesis. Only few CD41-positive cells co-expressed this transcription factor (Fig. 3a–d), and cells negative for both markers (CD41 and Gata2) were also observed (not shown in figures).

**Fig. 4:**
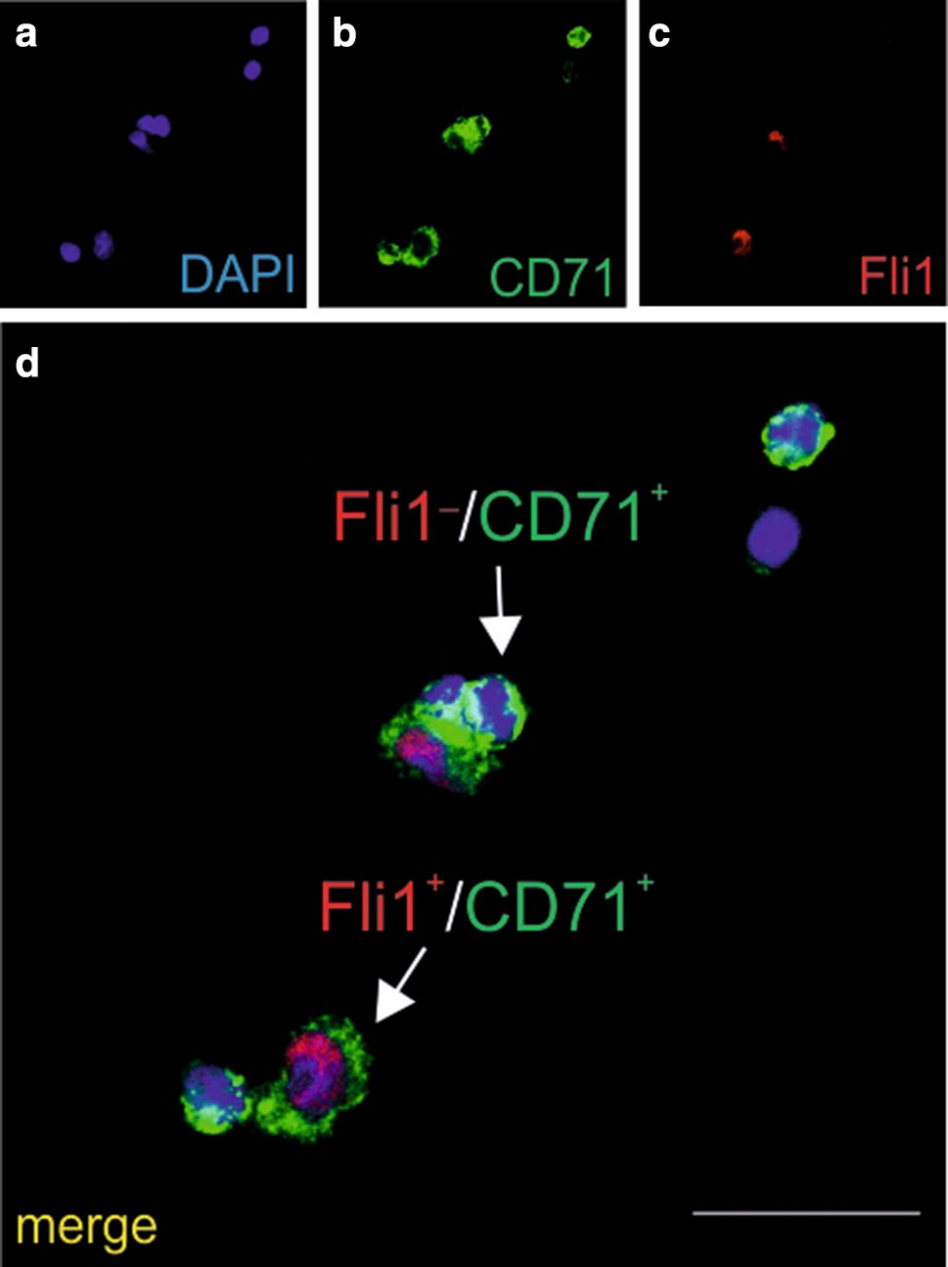
Cells growing in CFU-GEMM colonies express the CD71 and Fli1 markers. Confocal microscope images of smears prepared from CFU-GEMM colonies grown from CD31+/CD45−/CD71− cells stained with anti-CD71 (green) (**b, d**) and/or anti-Fli1 (red) (**c, d**) antibodies. Cell phenotypic diversity is demonstrated in merged images (**d**). CD71+/Fli1+ (red arrow) and CD71+/Fli1-(green arrow) cells are visible. Blue color in **a, d** marks cell nuclei counterstained with DAPI. Scale bar 25 µm. Lens magnification ×20, zoom ×6.5

In smears, cells with CD71^+^/Fli1^+^ and CD71^+^/Fli1^−^ phenotypes, which were indicative of their progression into erythrocytic (CD71^+^) or megakaryocytic (Fli1^+^) lineages, were observed, too (Fig. 4a–d).

**Fig. 5:**
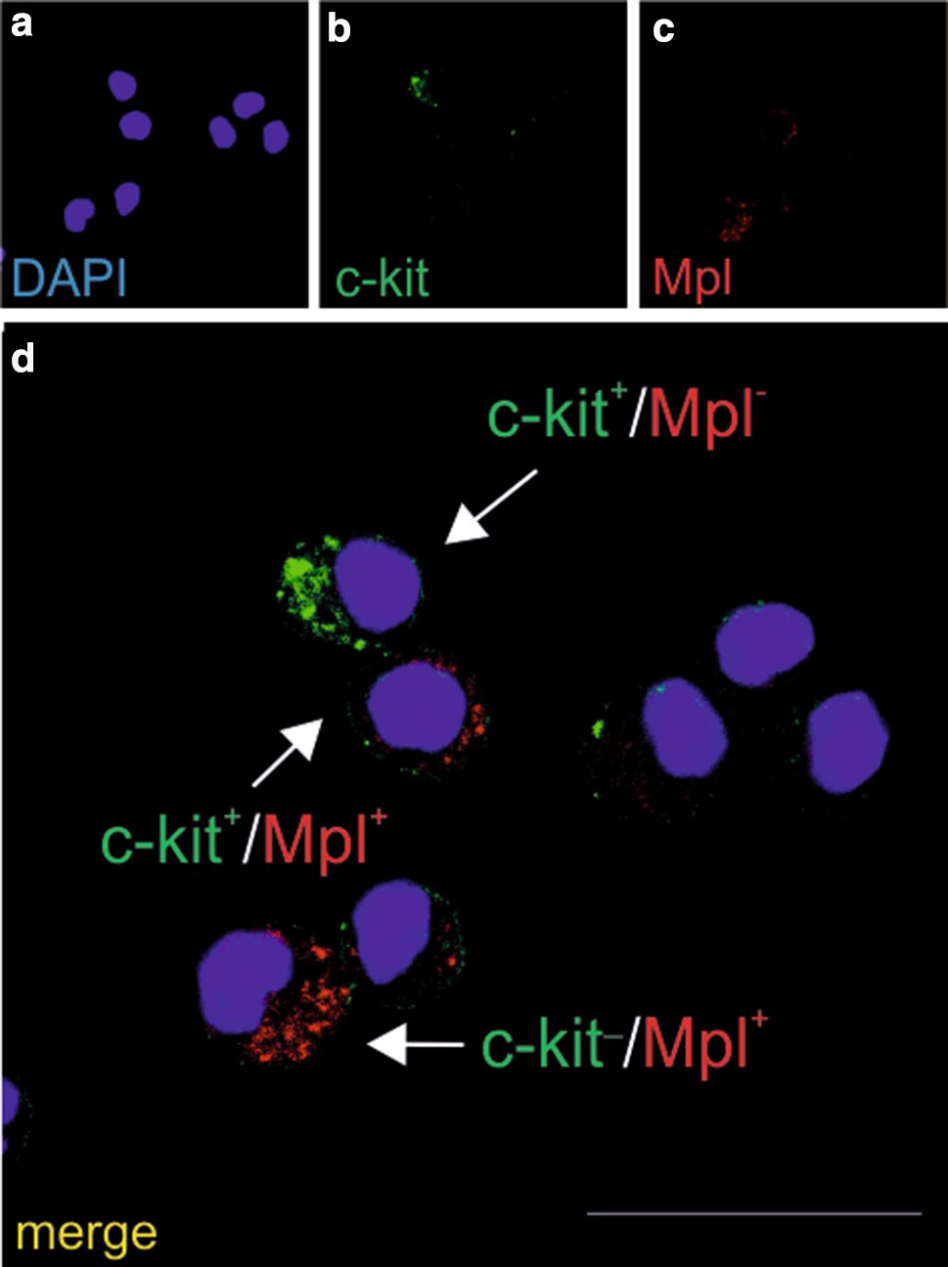
CFU-GEMM colonies give rise to cells expressing the c-kit and Mpl markers. Confocal microscope images of smears from CFU-GEMM colonies cultured from CD31+/CD45−/CD71−cells. Cells in the smears are stained with anti-c-kit (green) (**b, d**) and anti-Mpl (red) (**c, d**) antibodies. Cell phenotype diversity: c-kit+/Mpl+ (white arrow), c-kit-/Mpl+ (red arrow), and c-kit+/Mpl− (green arrow) is presented in panel d (merged images). Blue color in a and d marks cell nuclei counterstained with DAPI. Scale bar 25 µm. Lens magnification ×20; zoom ×7.6

The presence of another megakaryocytic lineage marker, Mpl (a thrombopoietin receptor), was observed in cells expressing or not expressing the c-kit molecule (a tyrosine kinase receptor expressed in hematopoietic stem cells and progenitor cells). However, it must be pointed out that some c-kit^+^ cells were Mpl^−^ (Fig. 5a–d).

**Fig. 6:**
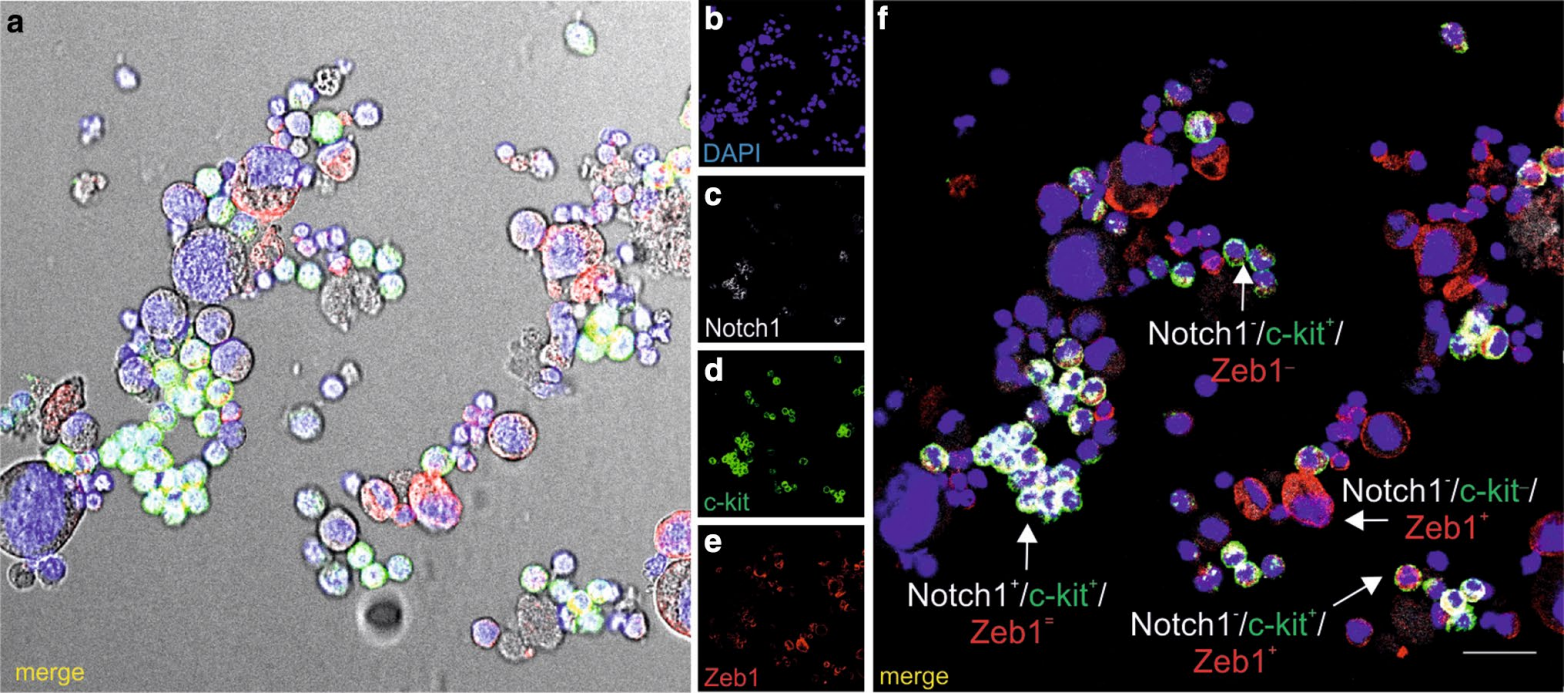
CD31+/CD45-/CD71 cells generate CFU-GEMM colonies with cells expressing Notch1, c-kit, and Zeb1 markers. Nomarski microscopy image (**a**) and confocal microscope images (other panels) of smears prepared from CFU-GEMM colonies. Cells are stained with anti-Notch1 (white) (**c, f**), anti-c-kit (green) (**d, f**), and anti-Zeb1 (red) (**e, f**) antibodies. Cells grown in colonies exhibit a diversity of marker expression (merged images in **f**). Notch+/c-kit+/Zeb1+ (dotted white arrow), Notch+/c-kit+/Zeb1− (white arrow), Notch−/c-kit+/Zeb1+ (yellow arrow), Notch-/c-kit+/Zeb1− (green arrow), Notch−/c-kit−/Zeb1+ (red arrow) cells are demonstrated. Cell nuclei are counterstained with DAPI (blue) (**b, f**). Scale bar 25 µm. Lens magnification ×20; zoom ×3.60

In some cells, c-kit was co-expressed with Zeb1, which is a transcription factor that becomes activated during epithelial-to-mesenchymal transition (EMT). Expression of these two markers varied in cells, as c-kit^+^/Zeb1^−^, c-kit^−^/Zeb1^+^, and c-kit^−^/Zeb1^−^ cells were observed as well. Some of the c-kit^+^ cells were also positive for Notch1 marker. However, only some of those double-positive cells (c-kit^+^/Notch1^+^) also co-expressed Zeb1 (Fig. 6a–f).

Similar cell phenotypes were observed in cell smears from CFU-GEMM colonies, which were derived from Flk1^+^/CD31^−^/CD45^−^/CD71^−^ cell population.

**Fig. 7:**
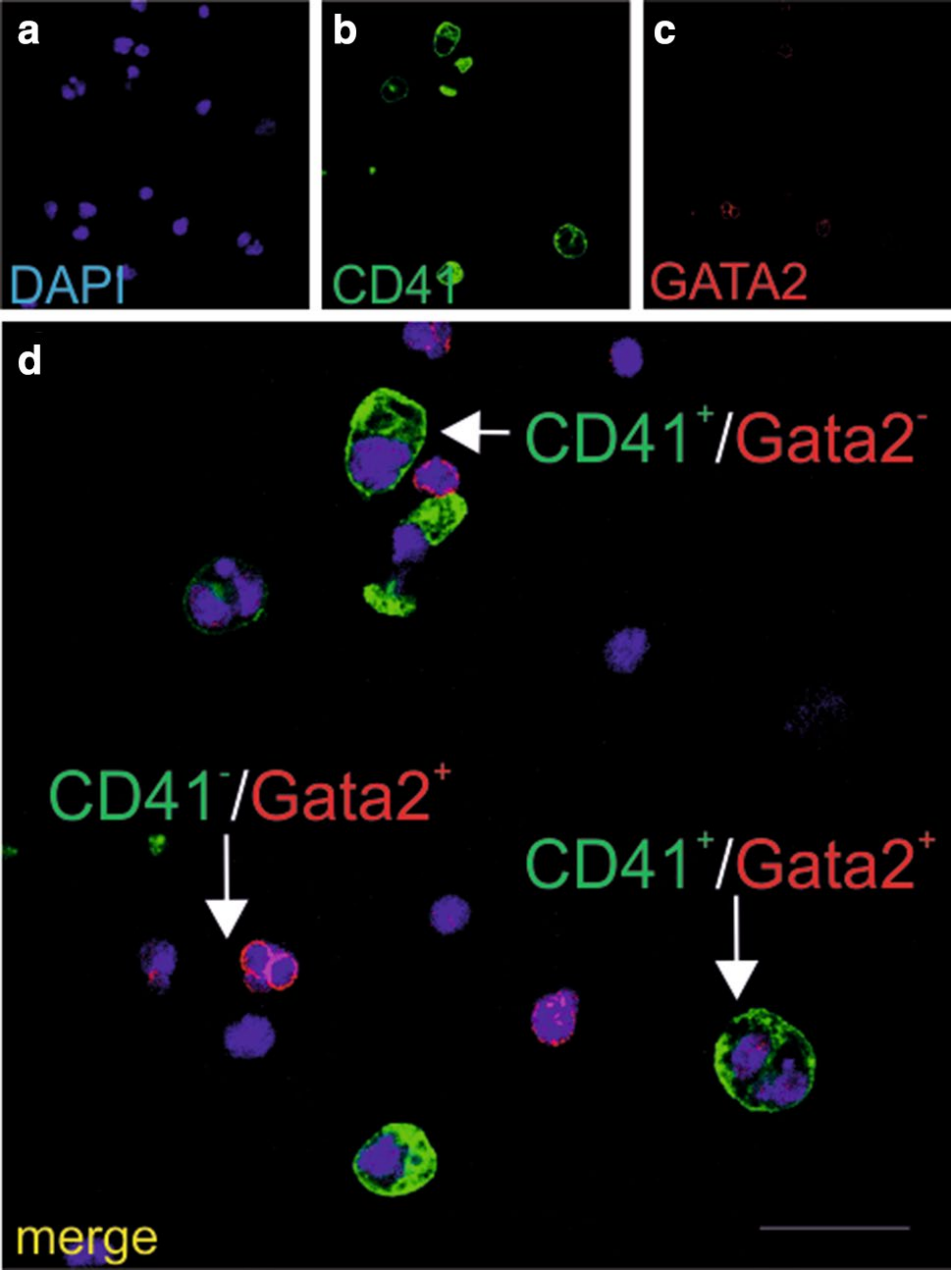
Cells growing in CFU-GM colonies express the CD41 and Gata2 markers. Confocal microscope images of smears prepared from CFU-GM colonies grown from CD31+/CD45−/CD71− cells stained with anti-CD41 (green) (**b, d**) and/or anti-Gata2 (red) (**c, d**) antibodies. Merged images (**d**) demonstrate phenotypic diversity: CD41+/Gata2+ (white arrow), CD41−/Gata2+ (red arrow), and CD41+/Gata2− (green arrow) cells are shown. Blue color in **a, d** marks cell nuclei stained with DAPI. Scale bar 25 µm. Lens magnification ×20; zoom ×6.8

Colonies of CFU-GM type were present after a 10-day culture of isolated CD31^+^/CD45^−^/CD71^−^, Flk1^+^/CD31^−^/CD45^−^/CD71^−^, and CD45^+^ and/or CD71^+^ cell populations. Cells from this type of colonies were characterized by various combinations of CD41 and/or Gata2 expression: CD41^+^/Gata2^+^, CD41^+^/Gata2^−^, CD41^−^/Gata2^+^, and CD41^−^/Gata2^−^ (Fig. 7a–d).

**Fig. 8:**
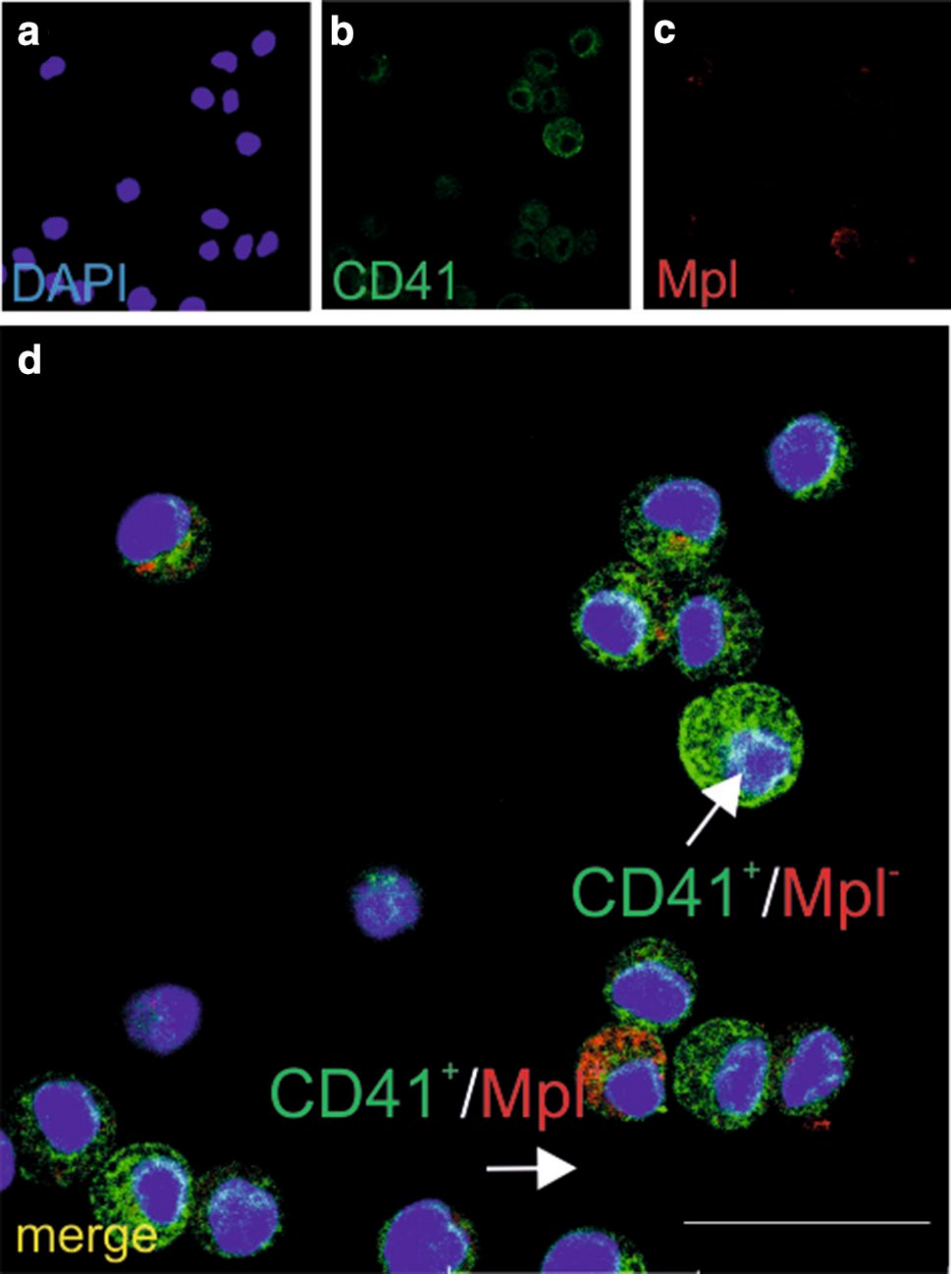
CD31+/CD45−/CD71− cells generate CFU-E colonies with cells expressing CD41 and Mpl markers. Confocal microscope images of smears prepared from CFU-E colonies stained with anti-CD41 (green) (**b, d**) and/or anti-Mpl (red) (**c, d**) antibodies. Merged images **d** show CD41/Mpl double-positive (red arrow) and CD41+/Mpl− (green arrow) cells. Nuclei are counterstained with DAPI (blue) (**a, d**). Scale bar 25 µm. Lens magnification ×20; zoom ×7.0

CFU-E-type colonies emerged from CD31^+^/CD45^−^/CD71^−^ and CD45^+^, and/or CD71^+^ cell subpopulations isolated from PE. In smears, cells from these colonies expressed both CD41 and Mpl markers, or were negative for Mpl (Fig. 8a–d).

**Fig. 9:**
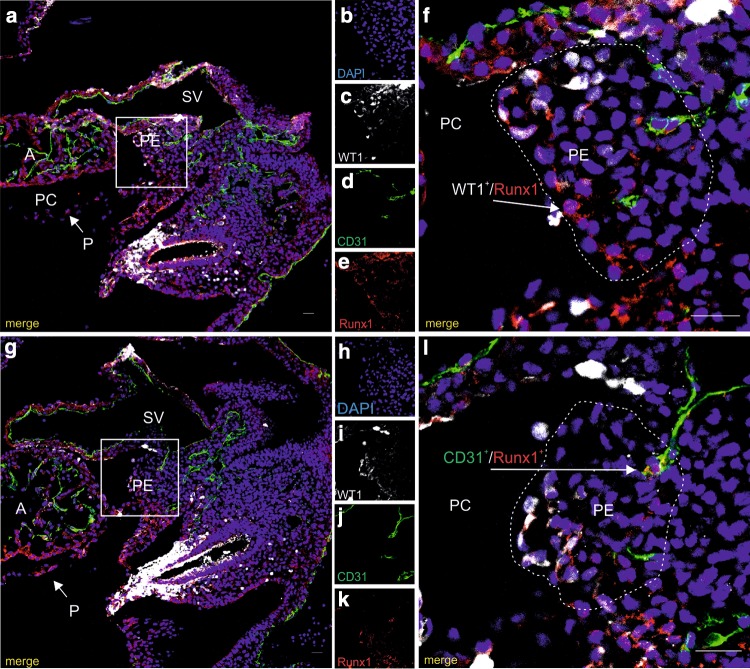
Runx1 + cells are scattered in the superficial region of the PE and in the endothelial lining of proepicardial capillaries. Confocal microscopic panel (**a**–**l**) demonstrating sections of a 9.5-dpc embryo stained with WT1 (white) (**a, c, f, g, i, l**), anti-CD31 (green) (**a, d, f, g, j, l**), and anti-Runx1 (red) (**a, e, f, g, k, l**) antibodies. Cell nuclei are counterstained with DAPI (blue) (**a, b, f, g, h, l**). The areas boxed in a and g are enlarged in f and l, respectively. A Runx1-positive cell co-expresses the WT1 marker (**f**) and localizes at superficial region of the PE (arrow), while another Runx1+ cell co-expressing CD31 (**l**) is the constituent of a proepicardial capillary (arrow). Dotted line borders the PE (**f, l**). *PC* pericardial cavity, *A* atrium, *SV* sinus venosus, *PE* proepicardium, **P** pericardium. Scale bars 25 µm. Lens magnification ×20; zoom ×3.0 (**f, l**)

In frozen sections of whole embryos, the PE was identified on the basis of its anatomical location and a positive immunostaining for a specific marker, WT1 (Fig. 9a–l). This marker was especially evident in cells located on the surface of the PE. Some of WT1-positive cells co-expressed the transcription factor Runx1 (Fig. 9a–f). Runx1 was rarely co-localized with the CD31 molecule, which marked cells in the wall of blood capillaries (Fig. 9g–l).

**Fig. 10:**
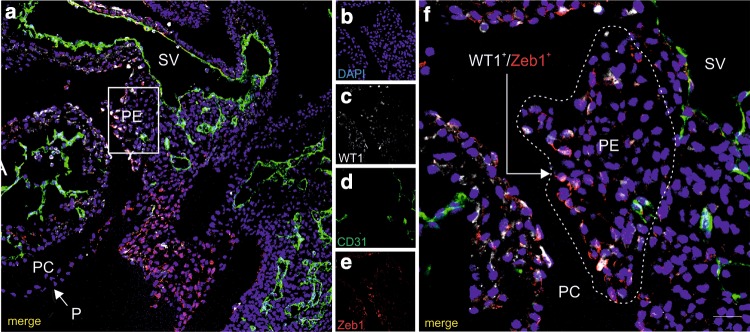
Zeb1 marker is expressed by some proepicardial cells. Confocal microscope images of a 9.5-dpc embryo section (**a**–**f**). Cells are stained with anti-WT1 (white) (**a, c, f**), anti-CD31 (green) (**a, d, f**), and anti-Zeb1 (red) (**a, e, f**) antibodies. Merged images (**a, f**) include DAPI-stained cell nuclei (blue). The area of PE boxed in a is enlarged in f. The PE is bordered with a dotted line (**f**). WT1 + cells located close to the proepicardial surface co-express Zeb1 (arrow in **f**). *PC* pericardial cavity, *A* atrium, *SV* sinus venosus, *PE* proepicardium, *P* pericardium. Scale bars 25 µm. Lens magnification ×20; zoom ×2.8 (**f**)

WT1-positive cells were also positive for Zeb1, which was localized in the nucleus and in the cytoplasm of those cells (Fig. 10a–f). However, no co-localization of Zeb1 with CD31 marker was detected. A few cells located on the surface of epicardium were also Zeb1-positive.

**Fig. 11:**
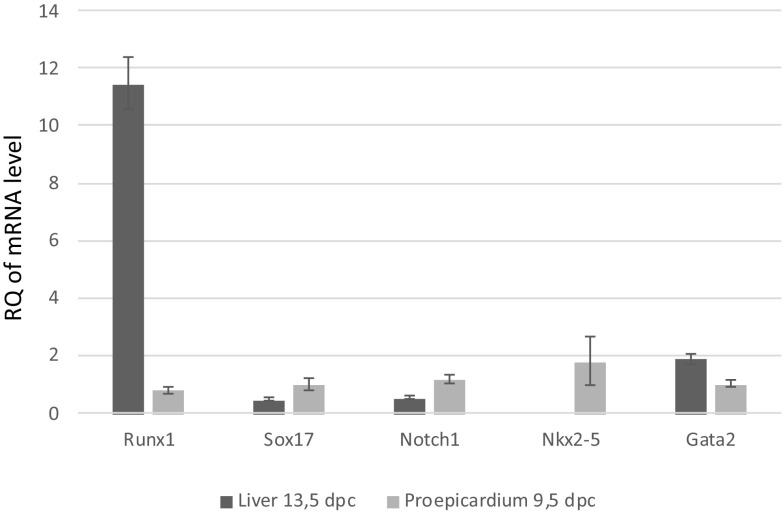
Results of RT-PCR analysis showing Runx1, Sox17, Notch1, Nkx2-5, and Gata2 expression in the PE of 9.5-dpc embryos and in the liver of 13.5-dpc embryos. Expression of Nkx2-5 occurs only in the liver. Asterisks indicate statistically significant differences (*p* < 0.05)

Real-Time RT-PCR analysis of mRNA for Runx1, Sox17, Notch1, Nkx2-5, and Gata2 demonstrated differences in the expression level of these markers in the PE at 9.5 dpc, and in the liver of 13.5 dpc embryos. PE cells expressed all those mRNAs, while in the fetal liver, the expression of Nkx2-5 was absent (Fig. 11). The mRNA expression levels for Runx1 and Gata2 were significantly higher in the liver as compared to the PE. On the other hand, the level of mRNA for Notch1 was significantly higher in the PE than in the fetal liver.

## Discussion

In this study, we showed for the first time that specific-cell populations isolated from mouse PE have a hemogenic potential as assessed by a colony-forming cell assay, immunohistochemical features of cells grown in colonies, and mRNA expression levels for selected genes involved in HE activity. Our finding is consistent with study by Tomanek et al. performed in quail embryos. The authors, using retroviral cell tagging of the proepicardium, demonstrated that some erythroblasts/erythrocytes located in blood islands and small vascular tubes were progeny of proepicardially derived hemangioblasts (Tomanek et al. 2006). Although this study had some limitations discussed by authors, our results confirm such possibility of hemangioblast origin from the PE, albeit in different species. Moreover, we characterized these cells *in situ* by immunoconfocal microscopy demonstrating the expression of Runx1 antigen, and also showing cell colonies of various markers typical for hematopoietic lineages that derive from PE endothelial cells. In addition, we performed RT-PCR study demonstrating an elevated message for genes crucial for hematopoetic cell emergence.

The CD31^+^/CD45^−^/CD71^−^ cell population had the highest potential to form hematopoietic colonies. Moreover, this cell population formed the most heterogenic type of colonies. The CD31 molecule is a marker of EC (Newman 1997). In the PE, EC are of various origin (Cossette and Misra 2011) and form a continuous network of vascular tubules connected with the sinus venosus endothelium (Niderla-Bielinska et al. 2015). It is well known that a subpopulation of EC, referred to as the hemogenic endothelium, has a hemogenic potential (Jaffredo et al. 1998; Boisset et al. 2010). This specific EC subpopulation forms a transient cell type, which is estimated to constitute between 1 and 3% of the entire endothelial cell population in murine yolk sac and the aorta/gonad/mesonephros region (Gritz and Hirschi 2016). By means of clonal ex vivo assays (in which endothelial cells isolated from the mid-gestation aorta and vitelline and umbilical arteries were co-cultured on supportive stroma), it has been documented that only about 0.1% (9.5 dpc), 1.3% (10.5 dpc), and 0.29% (11.5 dpc) of the entire endothelium represents a functional hemogenic endothelium (Ganuza et al. 2017). Here, we present the evidence that some EC of the PE also possess the hemogenic potential. The low number of colonies obtained in the in vitro colony-forming cell assay can be explained by the fact that the EC population that is located in the PE and exhibits hemogenic potential is also very small.

It has been postulated that the HE is formed from the arterial type of endothelium rather than from the venous type (Lizama et al. 2015). On the other hand, Yzaguirre and Speck reported that hematopoietic clusters were developing in both the arterial and venous plexi of the yolk sac from 9.5 dpc to 10.5 dpc (Yzaguirre and Speck 2016). Our previous study demonstrated that the PE capillary network was lined with immature EC (Niderla-Bielinska et al. 2015). Thus, it seems possible that EHT may occur also at early stages of EC differentiation and before they differentiate into the arterial or venous type. It was shown that EC have a high plasticity (Dejana et al. 2017). Therefore, it is likely that immature EC may exhibit hemogenic potential in vitro. Since no scattered CD31^+^ cells had been found in murine PE (Niderla-Bielinska et al. 2015), we presume that the CD31-positive cells isolated and cultured in this study were derived entirely from proepicardial capillaries and represented EC.

The highest number of colonies obtained after 10 days of culture was of CFU-GEMM type, in which there was a population of CD31^+^/CD41^+^ cells. It has been documented that this phenotype is characteristic for cells undergoing EHT (Mikkola et al. 2003). Therefore, it is possible that CD31/CD41-double-positive cells represent a specific fraction of endothelial cells which possess a hemogenic potential.

We had expected that CD41^+^ cells, present in our CFU-GEMM colonies grown from CD31^+^/CD45^−^/CD71^−^ cells, would co-express the Gata2 transcription factor. This transcription factor was reported to be present in hematopoietic stem cells and progenitor cells, including early erythroid cells and megakaryocytes (Vicente et al. 2012). Surprisingly, we observed such co-expression only in some of the CD41-positive cells. We hypothesize that Gata2 expression might occur only during a limited, specific time window, and that in our study, at 10 days of culture, this transcription factor was already absent or had been downregulated in some cells. Moreover, it was shown that not all hematopoietic progenitor cells in murine aorta, vitelline and umbilical arteries, as well as in fetal liver, required or expressed Gata2 (Kaimakis et al. 2016) suggesting the existence of Gata2-independent hematopoietic progenitors (Kaimakis et al. 2016).

Cells present in the same CFU-GEMM colonies, cultivated from CD31^+^/CD45^−^/CD71^−^ population, possessed markers for erythroblastic (CD71^+^) and megakaryocytic (Fli1^+^) lineages (Marsee et al. 2010; Dong et al. 2011). These two markers were expressed in various cells in different combinations, and both double-positive (CD71^+^/Fli1^+^) or single-positive (CD71^+^/Fli1^−^, or CD71^−^/Fli1^+^) cells were present. This phenomenon may be interpreted as a sign that these cells were at different stages of differentiation, since erythrocytes and megakaryocytes arise from a common progenitor (Klimchenko et al. 2009).

In our study, the megakaryocytic lineage was additionally labeled with anti-Mpl antibodies. Mpl, the thrombopoietin receptor, is also co-expressed with the c-kit molecule, which is characteristic of hematopoietic stem cells and/or progenitor cells (Kimura et al. 1998; Ng et al. 2014).

The novel finding of this study is the detection of the co-expression of Zeb1 in some c-kit-positive cells. Since Zeb1 is an important factor engaged in epithelial–mesenchymal transition (Wang et al. 2010; Zhang et al. 2016), we believe that this transition may play a role in the pathway of our cells: from EC to cells with hemogenic potential. These c-kit/Zeb1 double-positive cells frequently co-expressed Notch1, another factor essential for HE specification. It is known that Gata2, which is expressed in the endothelium and in budding hematopoietic clusters within the dorsal aorta, is a transcriptional target for Notch1 (Robert-Moreno et al. 2008; Tober et al. 2016).

Cells of all the phenotypes described above, characteristic for CFU-GEMM colonies and cultivated from CD31^+^/CD45^−^/CD71^−^ cells, were also observed in the CFU-GEMM colonies which had differentiated from the Flk1^+^/CD31^−^/CD45^−^/CD71^−^ cell population.

The Flk1 molecule is expressed on EC alongside CD31, but it is also expressed in the endothelium at earlier stages of differentiation (Wu et al. 2003; Marcelo et al. 2013). Moreover, Flk1 is expressed in early mesodermal cells that give rise to hemangioblasts (Chung et al. 2002) and are considered common precursors of endothelial and hematopoietic cell lineages.

One should remember that mesodermal cells constitute an important component of the PE and form progenitors of various cell types (Jenkins et al. 2005). Isolated in this study, the Flk1^+^/CD31^−^/CD45^−^/CD71^−^ cell population may consist of cells less differentiated than the population bearing the CD31^+^/CD45^−^/CD71^−^ phenotype. Moreover, apart from giving rise to cells with similar phenotypes to those obtained from the CD31^+^/CD45^−^/CD71^−^ cells, the former cell population formed a lower number of colonies. We speculate that the degree to which cells are advanced in their development has some influence on their efficiency of in vitro colonies formation. We believe that cells which are more advanced in development are also more efficient in the expression of their hemogenic potential.

In addition, after 10 days of culture, both cell populations isolated from the PE, i.e., CD31^+^/CD45^−^/CD71^−^ and Flk1^+^/CD31^−^/CD45^−^/CD71^−^, formed colonies of CFU-GM type. Most of the cells forming those colonies expressed both CD41 and Gata2, or at least one of these molecules, and we believe that this demonstrates and confirms their hemogenic commitment.

The lowest number of colonies detected in our study was those of the CFU-E type. They emerged in in vitro cultures from CD31^+^/CD45^−^/CD71^−^, and CD45^+^ and/or CD71^+^ cells. Cells forming these colonies expressed CD41 and Mpl markers, which were co-localized or expressed separately. This suggests that by the 10th day of culture the CFU-E colonies were at an early stage of differentiation as some cells in these colonies still retained the megakaryocytic marker Mpl.

The growth of colonies of the CFU-E type should be expected from CD45^+^ and/or CD71^+^ cell populations. It is because these cells already have some hemogenic potential as they express antigens such as CD45 and CD71, which are typical common leukocyte antigen and erythroblastic markers, respectively (Nakano et al. 1990; Marsee et al. 2010). These isolated CD45^+^ and/or CD71^+^ cell populations might have developed from CD31^+^/CD45^−^/CD71^−^, Flk1^+^/CD31^−^/CD45^−^/CD71^−^, or some other cells which resided in or migrated to the PE from its surroundings. Since the presence of erythroblasts in the lumen of PE capillaries had been observed previously (Niderla-Bielinska et al. 2015), it cannot be excluded that some of the cells in our CD45^+^ and/or CD71^+^ population had arisen from those cells, which had been entrapped within the lumen of these capillaries. Others have been reported that CD45^+^ hematopoietic precursors accumulated on the developing quail heart surface preceded association of these cells with vasculature, suggesting hematopoietic commitment precedes formation of blood island in the coronary vasculature (Kattan et al. 2004). Since these CD45^+^ cells appeared on the developing heart surface at the same time and in a similar location as QH-1-positive endothelial cells (the endothelial-specific antibody in quail) the latter being detected in the proepicardial organ at developmental stage in which PE had contacted the heart, these authors speculated that both cell types derived from the same structure, namely, PE. On the other hand, our previous study demonstrated some CD45^+^ cells expressing the Lyve1 antigen located in the subepicardium of mouse heart what suggests their macrophage identity (Jankowska-Steifer et al. 2015). Therefore, it cannot be excluded that at least some of CD45^+^ cells, described previously by Kattan et al. in the embryonic avian subepicardium, are of macrophage type (Kattan et al. 2004).

The variability of colonies grown from specific-cell-type populations isolated from the PE in our study may reflect the HE heterogeneity, which had been suggested by other authors (Ganuza et al. 2017).

The demonstration that PE-derived cell populations possess hematopoietic potential in vitro raises the question of these cells’ identity, because isolating from an embryo as small structure as the PE may be tricky and may create technical problems. Therefore, for the PE preparation, we used a technique which had been previously described in detail by others (Garriock et al. 2014). Moreover, apart from using PE’s anatomical position as a criterion for its localization, we immunostained frozen sections of whole embryos with a PE-specific marker, the WT1 (Armstrong et al. 1993), together with the CD31 molecule. It has been known that definitive hematopoiesis arises from the HE (Hirschi 2012). Since the PE is endowed with its own blood vessels, we aimed to detect CD31-positive cells which co-expressed Runx1, a transcription factor essential for regulation of definitive hematopoiesis. It must be pointed out that only primitive hematopoiesis in the yolk sac and definitive hematopoiesis in hemogenic endocardium in the heart at 8.5–9.5 dpc proceed independently of Runx1 expression, and in all other anatomical locations, hematopoiesis requires its expression (Nakano et al. 2013).

Interestingly, in our in situ immunostaining study, only few cells expressed both Runx1 and CD31 molecule, and these cells were situated in the wall of blood vessels. This indicates that only a limited number of EC in the PE are able to transform into cells having hemogenic potential. Such observation is consistent with quantitative evaluations, performed by other authors, for the HE found in various locations (Ganuza et al. 2017). Our detecting of CD31/Runx1-double-positive cells may suggest the presence of the HE in the PE. The PE is speculated to be a source of dormant cells with a hematopoietic potential rather than a place of active hematopoiesis. Endothelial cells which form blood vessels in the PE are probably of the venous type and venous origin, because these blood vessels were found to drain directly into the sinus venosus and have a venous phenotype (Lyve-1^+^) (Niderla-Bielinska et al. 2015). However, hemogenic potential was documented only for the yolk sac venous endothelium (Yzaguirre and Speck 2016). On the other hand, we previously postulated that endothelial cells of the PE were immature (Niderla-Bielinska et al. 2015). It is probable that such immature endothelium has a higher plasticity, which is related to the endothelium’s hemogenic potential.

Some of the WT1-positive cells also bore the Runx1 transcription factor, which is important in hemogenic specification and is critically required for EHT. Thus, this in situ observation also confirmed the existence of cells with hemogenic potential within the PE. Runx1^+^ cells, co-expressing the WT1 and devoid of the CD31 molecules, which were sporadically found in our material close to the surface of the PE, may have hematopoietic potential. After migrating to the surface of the developing heart, these cells may participate in generation of epicardium-derived cells (EPDCs). It cannot be excluded that Runx1^+^/WT1^+^/CD31^−^ cells play a role in coronary plexus formation after differentiating into the epicardium and EPDCs. It has been reported that Runx1 is required for the proper formation of coronary vessels during cardiogenesis, and that Runx1-null mice have an underdeveloped coronary plexus in comparison with the wild-type mice (Lluri et al. 2015). Moreover, blood islands observed in the subepicardium arise from the endocardium (Red-Horse et al. 2010), and are associated with Runx1 hematopoietic cells (Yzaguirre and Speck 2016). For this reason, we speculate that some EPDCs participate in the formation of these blood islands.

In addition, by means of RT-PCR, we documented the expression of Runx1 mRNA in the PE cells. Using the same technique, we detected the expression of mRNAs for factors characteristic for the HE: Gata2, Notch1, Sox17, and Nkx2-5.

Notch1, after binding its ligand Jagged1, is responsible for HE specification and acts upstream of Runx1. Later, Notch1 is downregulated during hematopoietic cell generation. The expression of Notch1 may also be related to the acquirement of the arterial identity by endothelial cells.

Other authors did not find the expression and/or activity of Notch1 molecule in the PE (del Monte et al. 2011). The evidence of Notch1 activity, found in the epicardium at 11.5 dpc, was restricted to EPDCs (del Monte et al. 2011). In contrast to detecting the presence of Notch1 protein, we only found the expression of its mRNA in the PE, together with the expression of mRNA for Gata2 (a direct transcriptional target for Notch1 signaling). Recent experiments in zebrafish support the hypothesis that a major function of endothelial Gata2 is to upregulate Runx1 expression (Butko et al. 2015).

Sox17, a transcription factor acting upstream of the Notch1 (Clarke et al. 2013), is known as a regulator of endoderm and hematopoietic differentiation. It is selectively expressed in arterial endothelial cells in the mouse embryo. Tissue-specific, temporally controlled loss of arterial genes expression—Sox17 and Notch1—during EHT results in an increased production of hematopoietic cells. This formation of hematopoietic cells results from loss of Sox17-mediated repression of hematopoietic transcription factors, Runx1 and Gata2 (Lizama et al. 2015).

Therefore, a novel finding of our study is the demonstration in the PE of the expression of mRNA for these transcription factors, which may confirm a hemogenic potential of the CD31^+^/CD45^−^/CD71^−^ and Flk-1^+^/CD31^−^/CD45^−^/CD71^−^ PE cell populations.

A high level of mRNA expression for Nkx2-5 in the PE is intriguing, since this factor had been described originally as crucial for cardiac development and specification (Akazawa and Komuro 2005). The Nkx2-5 is also required for PE development, since cells of lateral plate mesoderm, which are precursors of the PE, express this transcription factor (Zhou et al. 2008). Nevertheless, it has been recently suggested that Nkx2-5 plays a role in hemoangiogenic lineage specification and diversification by initiating the hemogenic program. It was demonstrated that Nkx2-5^+^ cell progenitors contribute to the HE of the endocardium, and it was shown that in the AGM region they participate in blood cells formation (Zamir et al. 2017).

Therefore, it cannot be excluded that Nkx2-5 expression detected in our study may contribute to in vitro hemogenic activity of PE-derived cell populations.

Because Zeb1 is a transcription factor engaged in EMT, the expression of Zeb1 in the majority of WT1^+^ PE cells may indicate that these cells are committed to EMT in further steps of development (Liu et al. 2008; Zhang et al. 2015).

Precise roles of Zeb1 in hematopoietic development remain unclear; therefore, it is difficult to explain the lack of a co-localization of Zeb1 with the CD31 marker. We presume that the expression of these two markers in EC might not overlap, with the Zeb1 being present only within a very limited time window, and for this reason not being detectable in CD31^+^ cells at the time, we were evaluating the PE.

In conclusion, our major and novel finding is that cells isolated by us from the mammalian (mouse) PE and bearing the CD31^+^/CD45^−^/CD71^−^, the Flk-1^+^/CD31^−^/CD45^−^/CD71^−^, as well as the CD45^+^ and/or CD71^+^ phenotypes possess a hematopoietic potential in vitro, as assessed by a colony-forming cell (CFC) assay, and by in situ immunohistochemical labeling of cell lineages cultured in colonies. We also found that mouse PE cells express mRNA for Runx1, Notch1, Gata2, Sox17, and Nkx2-5, which are the key factors involved in activity of hemogenic endothelium.

## Electronic supplementary material

Below is the link to the electronic supplementary material.

Supplementary material 1 (TIF 7343 KB)

Supplementary material 2 (TIF 7369 KB)
